# Traits of a mussel transmissible cancer are reminiscent of a parasitic life style

**DOI:** 10.1038/s41598-021-03598-w

**Published:** 2021-12-16

**Authors:** E. A. V. Burioli, M. Hammel, N. Bierne, F. Thomas, M. Houssin, D. Destoumieux-Garzón, G. M. Charrière

**Affiliations:** 1 IHPE, Univ Montpellier, CNRS, IFREMER, Univ Perpignan Via Domitia, Montpellier, France; 2 ISEM, Univ Montpellier, CNRS, EPHE, IRD, Montpellier, France; 3 CREEC/CANECEV (CREES), MIVEGEC, Unité Mixte de Recherches, IRD 224–CNRS 5290–Université de Montpellier, Montpellier, France; 4 LABÉO, Caen, France; 5 Normandie Université, Université de Caen Normandie, FRE BOREA, CNRS-2030, IRD-207, MNHN, UPMC, UCN, Caen, France

**Keywords:** Cancer, Cell biology, Ecology, Diseases

## Abstract

Some cancers have evolved the ability to spread from host to host by transmission of cancerous cells. These rare biological entities can be considered parasites with a host-related genome. Still, we know little about their specific adaptation to a parasitic lifestyle. MtrBTN2 is one of the few lineages of transmissible cancers known in the animal kingdom. Reported worldwide, MtrBTN2 infects marine mussels. We isolated MtrBTN2 cells circulating in the hemolymph of cancerous mussels and investigated their phenotypic traits. We found that MtrBTN2 cells had remarkable survival capacities in seawater, much higher than normal hemocytes. With almost 100% cell survival over three days, they increase significantly their chances to infect neighboring hosts. MtrBTN2 also triggered an aggressive cancerous process: proliferation in mussels was ~ 17 times higher than normal hemocytes (mean doubling time of ~ 3 days), thereby favoring a rapid increase of intra-host population size. MtrBTN2 appears to induce host castration, thereby favoring resources re-allocation to the parasites and increasing the host carrying capacity. Altogether, our results highlight a series of traits of MtrBTN2 consistent with a marine parasitic lifestyle that may have contributed to the success of its persistence and dissemination in different mussel populations across the globe.

## Introduction

Whether cancers belong to the host entity at the moment they start uncontrolled proliferation may be debated. Indeed, cancers could instead be considered as parasites having evolved from their host. In fact, after accumulating hundreds of mutations, the genome of cancerous cells diverges from the host genome with its own dynamics and genetic variation, becoming a new organism, composed of cheating cells, inside the multicellular host^1^. This organism is unable to survive autonomously without the host, from which it diverts the energy and resources necessary for its survival and growth, similarly to parasites^2,3^. But could it really be defined as a parasite?

Parasitism is defined as a form of symbiosis—an intimate relationship between two different species, in which the parasite cannot survive without its host while harming it in some way^4^. The parasitic lifestyle requires adaptations for several key events including between-host transmission and host colonization^5^. In order to survive, parasites have to be able to efficiently transmit between hosts, whereas cancers do not usually survive beyond the death of their original host. The parasitic status of classical cancers could be disputed on this basis —being non-contagious, the evolutionary life of classical cancers might be considered too short for them to be qualified as parasites.

However, some cancers have evolved the ability to transmit from host to host (see review^6^). Transmissible cancers are malignant cell lines, derived from a founder host, that have evolved the ability to spread from host to host by transmission of living cancerous cells. Indeed, they can be thought of as atypical parasitic life forms: parasites with a host-related genome. The transmissibility of these cancerous cell lines has been demonstrated through i) the presence of genetic chimerism in the diseased animals, with genetic differences between tumor and host cells, and (ii) the genetic similarity between tumors of different individuals. To date, several lineages of transmissible cancers have been described: one in dogs (Canine Transmissible Venereal Tumor, CTVT^7,8^, two in Tasmanian devils (Devil Facial Tumor Disease, DFT1 and 2^9,10^, and seven in various bivalve families (Bivalve Transmissible Neoplasia, BTN)^11–14^. Among BTNs, MtrBTN1 and MtrBTN2 are two distinct lineages with a *Mytilus trossulus* origin that infect mussel species of the genus *Mytilus*. MtrBTN2 has been observed in *M. trossulus*, *M. edulis*, *M. galloprovincialis*, and *M. chilensis*, showing that their transmission can cross the species barrier^12,13,15,16^. MtrBTN2 has been detected on several continents—South America, Asia, and Europe—^13,15–17^. It seems to have a fairly constant low prevalence among years and sites, indicating that it could be an endemic disease throughout the world. The low prevalence suggests that resident strains of hosts and parasites may have reached a population dynamic equilibrium, meaning that co-evolutionary processes are occurring.

Adaptations to a parasitic lifestyle have been supported by experimental data in Tasmanian devil and dog transmissible cancers, for which contact-dependent transmission routes have been characterized^18,19^. But because mussels are sessile animals, infectious agents must travel from host to host in the open seawater. Therefore, they must be able to survive outside their original host until they find a new host to infect. This trait is also of crucial importance for epidemiological predictions. Sunila and Farley^20^ reported that "sarcoma cells" from the soft-shell clam *Mya arenaria* were able to survive at least 48 h in seawater (Temperature = 24 °C, Salinity = 15 g/L). This cancer is very likely to be the same BTN lineage subsequently evidenced by Metzger et al.^11^, however, characterization of survival capacities in the outside-host environment is lacking for cancer cells unambiguously demonstrated to be transmissible.

Host colonization success depends also on the ability of parasites to proliferate in their hosts. BTNs are characterized by the proliferation of large, rounded, basophilic and polyploid cells with a high nucleus-to-cell ratio^21^ that can be found circulating in the hemolymph and invading vesicular connective tissues. Several histological studies indicated a significant amount of mitotic cells within the connective tissue, indicating that BTN cells actively proliferate^17,22^ although flow cytometry analyses of DNA content per cell have not revealed any significant signal of mitotic neoplastic cells circulating in the hemolymph^16,17^. Therefore accurate quantifications of BTN proliferation rate are still missing.

As a consequence of their lifestyle, parasites often have profound effect on the fitness of their hosts. When parasites harbor significant multiplication rates, the intra-host load that can be reached depends strongly on the host resources available for parasite population growth (i.e. host carrying capacity). An increased host carrying capacity can be achieved by manipulating host energetic resources to the parasite profit and by curbing the decrease in host viability. As a consequence, many different parasites can override the host’s resources allocation strategy by limiting the energy devoted to host reproduction. Castrator parasites annihilate the reproductive ability of their host to their own advantage, but they keep their host alive to ensure their own long life^23^. The abundant literature on bivalve "disseminated neoplasia"—which can be suspected to be BTN in most cases, even though genetic analyses were missing to confirm transmissibility—reported equivocal results concerning the effects of neoplasia on gametogenesis^24–29^. But we still ignore the consequences of MtrBTN2 invasion on the mussel energetic resources and reproductive cycle.

In the present work, we aimed to investigate the parasitic traits of MtrBTN2, by focusing on i) MtrBTN2 survival capacity in the environment, which is essential for the transmission and persistence in mussel populations over time; ii) MtrBTN2 proliferation rate within *M. edulis* host; iii) the cost of MtrBTN2 infection on the host fecundity.

Our results show that MrtBTN2 harbors phenotypic characteristics that make it apparently adapted to a parasitic lifestyle and indicate that MtrBTN2 may be considered as a castrator parasite.

## Results

Neoplastic cells belonging to the MtrBTN2 lineage were isolated from *Mytilus edulis* mussels farmed in Normandy (France). The prevalence of cancerous mussels was low in the sampling area (2.66%) with 39 positive individuals out of 1466, according to cytological observation of mussel hemolymph. Among the 39 cancerous mussels, 16 were also analyzed by histology (Supplementary Table S1). All were confirmed to carry cancerous cells in their tissues. As expected in the sampling area, these mussels were diagnosed to carry the MtrBTN2 lineage, being positive by qPCR for the EF1α-i3 and mtCR-D genetic markers developed in this study (see Materials and methods) and by Yonemitsu et al.^13^, respectively (Supplementary Table S1).

### MtrBTN2 cells survive several days in seawater

Surviving for few days in the outside-host marine environment is a prerequisite for MtrBTN2 cells to be able to contaminate new hosts. Therefore, we isolated MtrBTN2 cells from diseased mussels and compared their survival in seawater to normal hemocytes isolated from healthy mussels.

**Figure 1:**
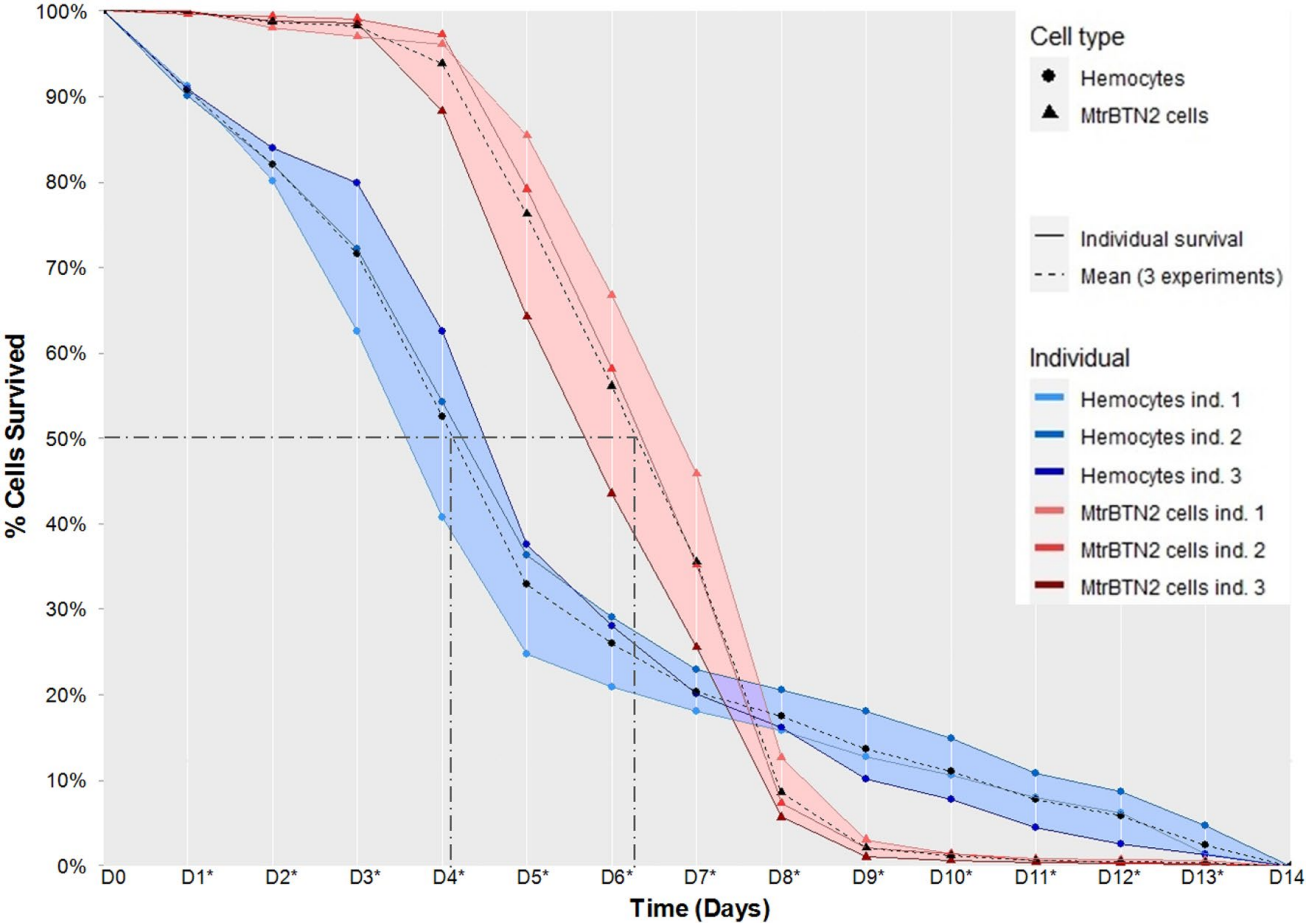
MtrBTN2 cells survive longer than hemocytes in seawater. The median survival rate was 6 days for cancerous cells and 4 days for hemocytes. Cells were seeded at a density of 2 × 10^4^ cells/well and incubated at 18 °C for 14 days. Warm colors correspond to MtrBTN2 cells and cool colors correspond to hemocytes. Dotted lines are the means of three independent experiments from different individuals, solid lines are the means of the three wells from each individual; asterisks on x-axis labels indicate significant differences between MtrBTN2 and hemocytes' survival (Mann–Whitney; D_1,_ U = 1, *P* = 0.0005; D_2_, *P* = 0.0002; U = 5; D_3_, *P* = 0.00004, U = 0; D_4_, *P* = 0.00003, U = 0; D_5_, *P* = 0.00003, U = 0; D_6_, *P* = 0.0003, U = 0; D_7_, *P* = 0.0004, U = 0; D_8_, *P* = 0.0004, U = 81; D_9_, *P* = 0.0004, U = 81; D_10_, *P* = 0.0003, U = 81; D11, *P* = 0.002, U = 75; D_12_, *P* = 0.01, U = 69; D_13_, *P* = 0.03, U = 66).

MtrBTN2 cells survival rate in seawater was significantly higher than that of hemocytes over 7 days (Mann–Whitney, *P* < 0.05; daily test statistics are reported in the Fig. 1 legend) with a median survival time of MtrBTN2 cells equal to 6 days, as opposed to 4 days only for hemocytes. The trend in survival rates of MtrBTN2 and hemocytes was very reproducible across three independent experiments, each one performed with cells isolated from distinct individuals. The daily percentage of living MtrBTN2 cells was close to 100% over the first three days (Fig. 1). MtrBTN2 cell survival started to decrease at day 4 only, and no cells remained viable after day 9. In contrast, hemocytes started to die from the first day of the experiment. In a second phase (day 3–5), hemocyte survival decreased at approximately the same rate of MtrBTN2 (day 4–8). In a third phase, the remaining hemocyte populations died more slowly until no living hemocytes remain at day 14. Comparable survival profiles were obtained were observed hemocyte populations from *M. galloprovincialis* or *Crassostrea gigas,* indicating that bivalve species had little effect on hemocyte survival capacity (Supplementary Fig. S1).

### MtrBTN2 cells proliferate at a very high rate in vivo

**Figure 2:**
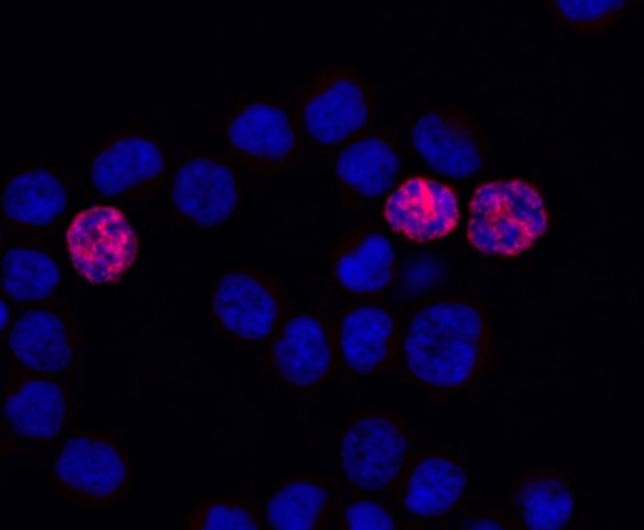
Labeling of MtrBTN2 cells with Click-iT EdU at the end of the in vivo EDU incorporation assay. Cells were withdrawn from the hemolymph and cytospun on a microscopy slide. EdU was labeled with Alexa Fluor 594 (red) and nuclei were counterstained with Hoechst 33,342 (blue). X400 magnification.

The ability of MtrBTN2 cells to proliferate in mussels was measured in vivo by performing EdU incorporation assays. EdU is a thymidine analog that is incorporated during DNA synthesis in mitotic cells. Cell proliferation was compared in 4 cancerous and 4 healthy mussels. MtrBTN2 cells were characterized by a high proliferation capacity, more than 17 times higher than that of normal hemocytes in healthy mussels, as observed in mussel hemolymph after a 9 h labeling time (Mann–Whitney, U = 16, *P* = 0.028). The proportion of circulating cells that underwent multiplication (cells having incorporated EdU in vivo) was 17.43% for MtrBTN2 (9.13 to 28.15%) and 0.99% for normal hemocytes (0.57 to 1.69%) (Table 1). A mean doubling time of about 74 h (~ 3 days) was calculated for MtrBTN2 cells from these results. Epifluorescence microscopy images of cells that have incorporated EdU in vivo are presented in Fig. 2.

**Table 1:** Cancerous mussels show high proliferation capacities. Multiplication rates are expressed as proportion of circulating cells having incorporated EdU in vivo 9 h after EDU injection*.* Cancerous and healthy mussels are compared, and all the MtrBTN2 cancerous individuals show significant higher multiplication rates (Mann–Whitney; U = 16, *P* = 0.028).

	Multiplication rate	
	Cancerous (%)	Healthy (%)
Mussel 1	9.30	0.57
Mussel 2	17.07	0.96
Mussel 3	15.20	0.72
Mussel 4	28.15	1.69
Mean	17.43	0.99

### MtrBTN2 affects mussel reproductive cycle

We next investigated the effect of MtrBTN2 on mussel physiology by histological analyses with a particular emphasis on reproductive cycle. For that we used cancerous and healthy mussels sampled from the field at various seasons. The reproductive status of all individuals was determined according to 4 histological stages (Table 2).

**Table 2:** Mussels infected by MtrBTN2 show poor gonadal development. Stage 0: resting; stage 1: gonia proliferation; stage 2: all gamete developmental stages present with only a few mature gametes; stage 3: predominance of ripe gametes; stage 4: resorption. I: undifferentiated gonad; F: female; M: male. The dominant stage is indicated in bold.

Gonadal stage	Nbr ind	Nbr ind./Sex			Nbr ind	Nbr ind./Sex		
		*U*	*F*	*M*		*U*	*F*	*M*
**Late January**								
**Cancerous**					**Healthy**			
Stage 0	N = 1	1	0	0	N = 0	–	–	–
Stage 1	**N = 3**	3	0	0	N = 0	–	–	–
Stage 2	N = 0	–	–	–	N = 0	–	–	–
Stage 3	N = 0	–	–	–	**N = 4**	0	2	2
Stage 4	N = 0	–	–	–	N = 0	–	–	–
**July**								
**Cancerous**					**Healthy**			
Stage 0	**N = 5**	5	0	0	N = 1	1	–	–
Stage 1	N = 0	–	–	–	N = 0	–	–	––
Stage 2	N = 0	–	–	–	N = 0	–	–	–
Stage 3	N = 2	0	1	1	N = 0	–	–	–
Stage 4	N = 0	–	–	–	**N = 5**	0	4	1
**December**								
**Cancerous**					**Healthy**			
Stage 0	**N = 5**	5	0	0	N = 0	–	–	–
Stage 1	N = 0	–	–	–	N = 2	2	0	0
Stage 2	N = 0	–	–	–	**N = 6**	0	4	2
Stage 3	N = 0	–	–	–	N = 0	–	–	–
Stage 4	N = 0	–	–	–	N = 0	–	–	–

**Figure 3:**
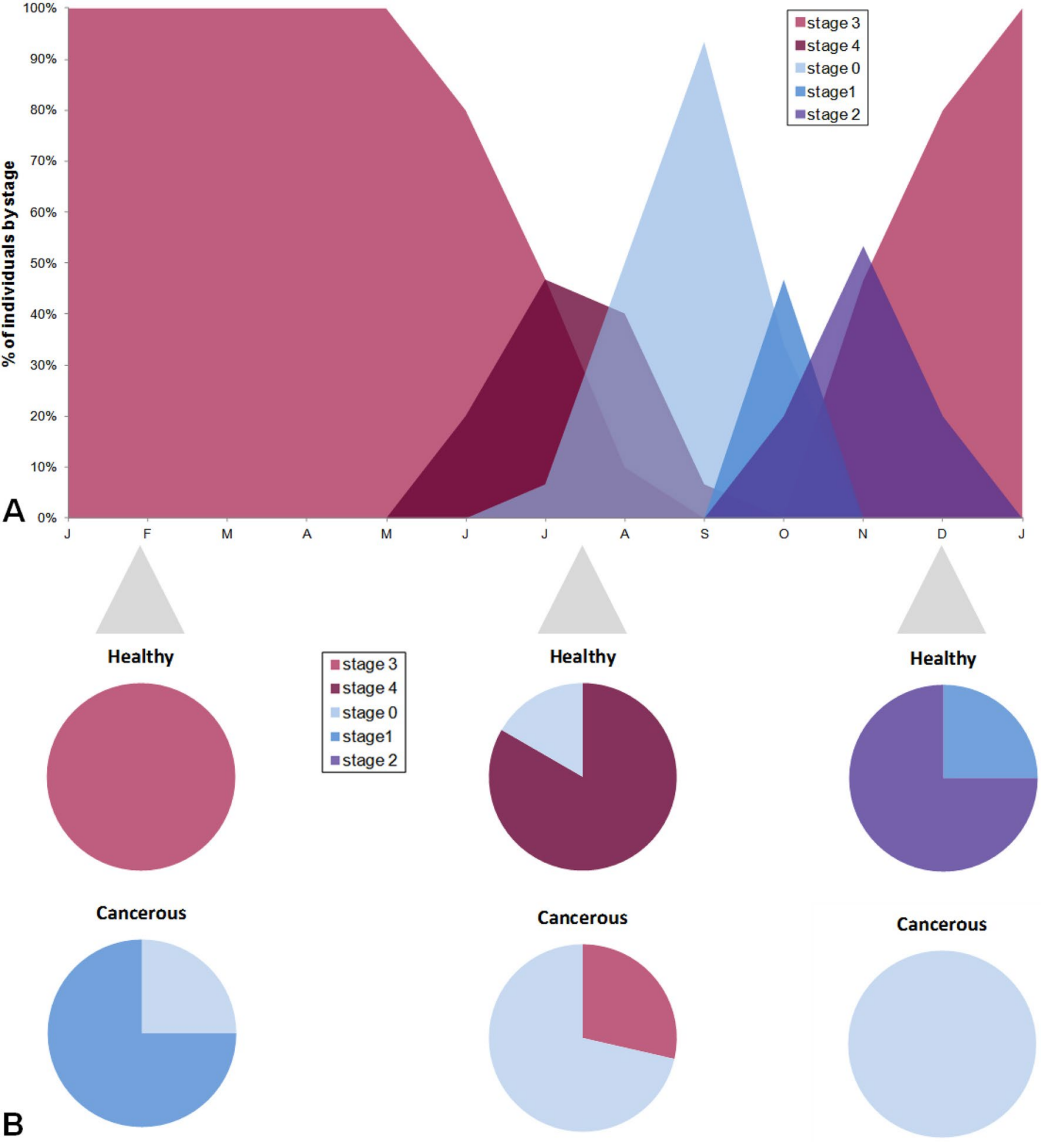
Gonadal development is delayed in MtrBTN2-affected (cancerous) mussels. (**A**) reference study in healthy Atlantic mussels (data from Randriananja^30^); (**B**) seasonal results from the present study comparing healthy and cancerous mussels. Stage 0: resting; stage 1, gonia proliferation; stage 2: all developmental stages present with only few mature gametes; stage 3: predominance of mature gametes; stage 4: resorption.

**Figure 4:**
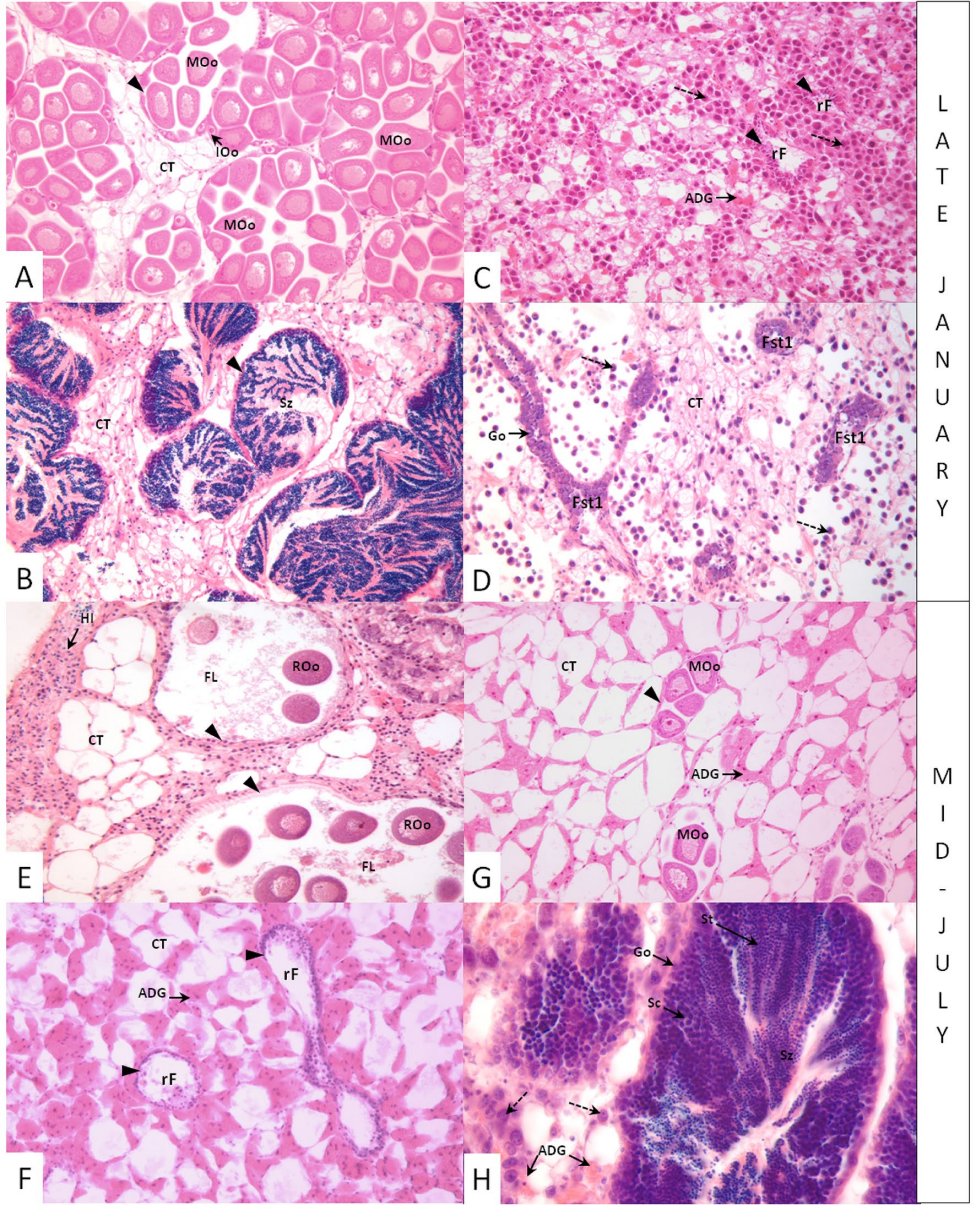
MtrBTN2 alters the gonadal development of *Mytilus edulis* and influences the amount of energetic resources. Histological micrographs of mussel gonads. Healthy mussels: (**A**, **B**, **E**, **F**). Cancerous mussels: (**C**, **D**, **G**, **H**). ADG: adipogranular cell; CT: connective tissue; FL: follicle lumen; Fst1: follicle stage 1; Go: gonia; IOo: Immature oocytes; MOo: mature oocytes; rF: resting follicle; ROo: residual oocyte; Sc: spermatocyte; St: spermatid; Sz: spermatozoa; arrow head: follicle wall; dotted arrows: cancerous cells. X200 magnification.

Most mussels infected with MtrBN2 (69%) were in a resting stage 0 (11 out of 16 mussels), all seasons being considered, as opposed to only 5.8% of healthy mussels (1 out of 17) (Table 2). Not only cancerous mussels poorly entered into reproduction, but when they did, the process was delayed compared to the seasonal reproduction cycle of healthy *M. edulis.* Indeed, only healthy mussels followed the expected cycle observed for animals from the Atlantic coast of France^30^ (Fig. 3A–B). In late January, all healthy individuals had ripe gametes typical of reproductive stage 3 (Fig. 4A,B), whereas mussels affected by MtrBTN2 were at resting stage 0 (Fig. 4C) or gonia proliferation (stage 1) only (Fig. 4D). In July, healthy individuals were mostly at resorption stage 4 (Fig. 4E) and resting stage 0 (Fig. 4F), indicating that the reproductive season had ended and that the reconstitution of the energy storage in adipogranular cells (ADG) within the connective tissue had started (stage 0). On the same date, no cancerous mussels were at resorption stage but two individuals had ripe gametes. One was a female, in the initial stage of neoplasia: no gametogenesis process was still in course in the follicle wall (Fig. 4G); the number of oocytes was low and the small follicle size indicated that no spawning had occurred. The second was a male at a mid-stage of neoplasia (Fig. 4H) and at a slightly earlier stage of gametogenesis than the female since various developmental stages were present even though mature spermatozoa were abundant. Interestingly, while ADG cells were absent in the connective tissues of healthy mussels at reproductive stages 3 (Fig. 4A and E), we observed numerous ADG cells in both cancerous individuals with mature gametes. These observations suggest that gametogenesis might be inhibited soon after infection by MtrBTN2. In December, gametogenesis was observed in healthy individuals while no gametogenesis was observed in cancerous mussels.

## Discussion

In this study, we identified phenotypic traits of cancerous cells that may explain MtrBTN2 successful transmission and persistence in host populations worldwide. These traits encompass their ability to persist in the outside host environment and to proliferate at high rate in diseased mussels. We also observed an early cancer-induced castration in the diseased host, probably favoring host carrying capacity and/or malignant cells’ proliferation.

Although the transmission process remains unknown, survival in open seawater is a prerequisite for BTNs to behave as parasites. We demonstrated here that 50% of the MtrBTN2 cells are still alive after 6 days in seawater as opposed to 4 days for hemocytes. Although we could not measure survival of *M. trossulus* hemocytes, as this mussel species is not present in France, we showed that hemocytes from 3 bivalve species (*M. edulis, M. galloprovincialis* and *Crassostrea gigas*) display similar survival in seawater. Therefore, MtrBTN2 cells survive better than hemocytes in seawater over 7 days, and this is not due to the species from which cancer cells originated but it is rather a cancer specific difference.

The particular survival capacity of MtrBTN2 cells in seawater is of crucial importance for disease epidemiology since after leaving the host, MtrBTN2 cells could be transported for more than six days by marine currents to neighboring mussel hosts. Seawater is a diffusive and dilutive media. The probability that an inoculate of a sufficient number of infective cancerous cells meet a new permissive host quickly decreases with time and distance. Cell dilution and host densities may therefore greatly limit long-distance transmission success, similar to external fecundation of broadcast spawners that decrease in effectiveness at scales above a few meters^31^. The interest for a non-motile marine parasite should not be to increase its survival indefinitely, but mainly during the first hours after release as observed here with MtrBTN2 cells, which showed no mortality during the first 3 days. For comparison, *Bonamia ostreae,* a protistan parasite of the European flat oyster with a direct life cycle, is viable at 70% after 2 days in seawater^32^. Mechanisms used by MtrBTN2 cells to survive for 3 days in a non-physiological and nutrient-poor environment like seawater will require further investigations to elucidate whether they harbor particular metabolic adaptations.

The capacity of MtrBTN2 cells to survive in a nutrient-poor environment raises the question whether it results from a specialization to a parasitic lifestyle allowing for transmission through open seawater, or whether, it was inherited from the tissular origin of the primary tumor that remains unknown so far. The MtrBTN2 cells’ death curve with one single phase of mortality (day 4–9) suggests a strong synchronicity of cancerous cells’ death, which is in accordance with the clonal theory of neoplastic cells^33,34^. On the contrary, the hemocyte death curve harbor a multi-modal rate of death with different slopes over the 3 successive phases. This is probably due to the heterogeneity of the hemocyte populations. In fact, at least 3 main cell types have been described in the hemolymph of bivalve mollusks^17,35–37^ and may have different survival capacities. It can be speculated that MtrBTN2 cells have derived from one hemocyte subpopulations.

We have shown here that MtrBTN2 is an aggressive cancerous process whose active proliferation in vivo favors a rapid increase of intra-host population size. We estimated a mean doubling time of about 3 days. For comparison, the shortest doubling times in vivo of leukemic blast cells in acute leukemias and solid tumors were estimated at 5–10 days^38–41^. Short doubling times were also described in eukaryotic marine microparasites such as *Perkinsus marinus* (3–7 days)^42^. Several histological studies have described a high mitotic rate of mussel cancers within the vesicular connective tissue^17,22^ but, according to the flow cytometry analyses of the DNA content, no proliferation occurs in the hemolymph^16,17^. A possible explanation is that cancerous cells proliferate in the connective tissue before reaching the hemolymph. This circulating stage may be of prime importance in terms of transmission. Indeed, there is a permanent cell exchange between hemolymph and intravalvular fluids in bivalves. Recently, Caza et al.^43^ showed that under temperature stress conditions, circulating hemocytes were able to leave the hemolymph to gain access to the intervalvular fluid before entering other mussels. Therefore, hemolymph is a likely route for the leakage of cancerous cells outside the infected host.

Our results show that MtrBTN2 seems to act as a parasitic castrator—a common adaptive strategy in marine animal parasites^44^. Although we cannot exclude the hypothesis that it could simply be a by-product of the infection with no adaptive value^45,46^, a possible reason is that MtrBTN2 uses energetic resources from the host to sustain such a high intra-host proliferation. Indeed, one way that parasite can manipulate their host to access resources is to interfere with reproductive traits by castration thus favoring the re-allocation to these resources to the parasites and increasing the host carrying capacity^23^. MtrBTN2 was found to infect both sexes. Although the very low prevalence of the disease limits this kind of study due to modest sample size, we nonetheless observed clear differences between neoplastic and healthy individuals in terms of gonadal development. Interestingly, the progression of gametogenesis appeared altered in all cancerous individuals. All but five of the MtrBTN2-affected individuals showed no reproductive activity at all, and gametogenesis was delayed in those five remaining individuals compared to healthy mussels. Two of these five individuals had ripe gametes. While healthy individuals at this stage of the reproductive cycle have low energy reserves and few or no ADG cells, the two neoplastic individuals had many ADG cells, corroborating the observations of Cremonte et al.^47^ for *M. chilensis* from southern Argentina affected by disseminated neoplasia. According to these observations, we can hypothesize that gametogenesis is blocked soon after infection by MtrBTN2, at the stage reached at this time, and that the cancerous parasite potentially has a manipulative role.

Theory postulates that cancer lineages and parasites able to manipulate phenotypic traits in the host in ways that favor their proliferation and/or transmission to novel hosts should achieve higher fitness and consequently be favored by selection. However, host manipulation by cancer cells has not been extensively studied until now, even though the existence of strategies manipulating the host and leading to increased tumor fitness have been hypothesized^48^. Our study unravels that MtrBTN2 behaves as a castrator parasite by manipulating mussel hosts reproductive cycle. A decrease in fecundity of diseased animals can possibly further alter host population dynamics^49^. However, the low prevalence observed in mussel populations suggests a limited impact on host demography overall, possibly because resistant hosts have been selected through time. To persist, the parasite must therefore evolve continuously to adapt to its co-evolving host population.

From our present work, MtrBTN2 harbors traits consistent with a marine parasitic lifestyle. The presence of MtrBTN2 year-round in mussel populations^50^ suggests that no resistant or latent phases of this parasite are necessary for the persistence of the parasite and that continuous transmission occurs. The ability of MtrBTN2 to survive in seawater is likely a key determinant of its efficient transmission and diffusion in mussel populations worldwide. A more comprehensive view of MtrBTN2 transmission will require additional research on the ways that infective cancer cells leave their host and enter a new host. Beyond key phenotypic traits for transmission, we found that MtrBTN2 has characteristics of various type of parasites: microparasites^51^-as they multiply at very high rate within the host, and castrator parasites^52^-as they seem to prevent host reproduction. According to these results, MtrBTN2 could be classified within unicellular parasitic diseases. Altogether, our results indicate that MtrBTN2 cancer cells behave like unicellular castrator parasites.

## Materials and methods

### Mussel origin and MtrBTN diagnostic

All *Mytilus edulis* mussels*,* used for survival and proliferation tests were collected in July 2020 from an aquaculture farm in Agon-Coutainville (Normandy, Channel, France). Mussels were acclimated for two weeks at 14 °C in filtered seawater supplemented with a microalga diet. Gonadal stage assessment of these individuals was carried out, as well as for additional mussels sampled in the same site in late January and early December.

The presence of circulating neoplastic cells and the disease stage were diagnosed by cytological examination according to Burioli et al.^17^. To confirm the MtrBTN2 diagnosis, we targeted two genetic markers, one mitochondrial *mtCR-D* (MtrBTN2-specific^13^) and one nuclear EF1 located on intron 3 (EF1α-i3) (*M. trossulus*-specific, newly designed and described below). Briefly, a piece of mantle and gill were fixed in 96% ethanol, and DNA extraction was accomplished with the Nucleomag 96 Tissue kit (MACHEREY–NAGEL). We followed the manufacturer protocol with modified volumes of some reagents: beads were diluted 2 × in water, 200 µL of MB3 and MB4, 300 µL of MB5, and 100 µL of MB6. For *mtCR-D* marker amplification, we used the primer pair and the two-step cycling stage protocol (95 °C for 15 s and 60 °C for 30 s, for 40 cycles) designed by Yonemitsu et al.^13^. For *EF1α-i3* qPCR development, we used sequences of Faure et al.^53^ and Bierne^54^ to design a primer pair that specifically amplifies *M. trossulus* (Supplementary Fig. S2). Table 3 presents information on qPCR primer pairs. For amplification, we used a three-step cycling stage protocol (95 °C for 10 s, 58 °C for 20 s, 72 °C for 25 s) for 40 cycles. We carried out both qPCRs using the sensiFAST SyBR No-ROX Kit (meridian BIOSCIENCE) and the LightCycler 480 Real-Time PCR System (ROCHE).

**Table 3:** Primer sequences used for qPCRs.

Locus	Primer	Amplicon length (bp)	Source
EF1α-i3	*EF1α-i3-F: 5'-GGTGATGCACACCACATTCATTTAG*	210	Newly designed
	*EF1α-i3-R: 5'-TCTGGATTTCCATGAATCGGGAC*		
mtCR-D	*MchmtAB-DF6: 5'-GTACTTCATTTCCTTGCCA*	60	Yonemitsu et al.^13^
	*MchmtAB-DR2: 5'-AAAAGACAGGTGGAAAGGGGT*		

### Neoplastic cell survival capabilities in the environment

For the experiment, we selected three advanced-stage neoplastic *Mytilus* (> 95% of cancerous cells in the hemolymph) and three healthy individuals. At D_0_ after acclimatization, mussels were anesthetized by bathing them for 30 min in a solution containing 50 g/L of magnesium chloride dissolved in 2:1 distilled water: sterile seawater^55^. We drew 100 µL of hemolymph from each individual with a 26G syringe containing 200 µL of Alsever solution to avoid cell aggregation. Cells were pelleted at 600 × g for 10 min at 14 °C and resuspended in sterile natural seawater (0.22 µm filtered and autoclaved) supplemented with penicillin/streptomycin and 1 nM SYTOX Green (invitrogen) to a concentration of 20,000 cells per mL. For each individual, we placed 1 mL of this cell suspension in five wells of a 24-well cell culture plate. Of these five wells, three were used as a triplicate for the individual cell survival tests and two were used as controls by the addition of Triton-X100 (0.1%) to kill cells by membrane permeabilization and to obtain the fluorescence value of 100% cell death. These controls were also used to verify that the fluorescence did not decrease during the experiment. The six plates (from three neoplastic and three healthy individuals) were covered and placed in a cell culture incubator at 18 °C with humidity maintained at 98%. The fluorescence value of each well was measured daily at 480 nm/520 nm with a M200 plate reader (TECAN) until D_14_, and for each individual we calculated the mean fluorescence of the three wells used as triplicates and of the two control wells (100% mortality). To determine the survival rates of cells from each individual, we compared these two mean fluorescence intensities and we converted the result to percentage of surviving cells. Finally, the survival rates of MtrBTN2 cells and hemocytes from healthy individuals were compared. The pH and osmolarity of the suspension were measured at T_0_ and at the end of the experiment. The pH value at D_0_ was 8.1 and decreased to a mean value of 7.9 (range 7.8–7.9) by D_14_. The osmolarity at D_0_ was 1151 mOsm/L and reached 1220 mOsm/L by the end of the experiment.

In order to verify whether hemocyte survival capacities in seawater could be species specific, we compared hemocytes’ surviving capacities from different bivalve species. We performed the supplementary survival tests with hemocytes from 2 different bivalve species, *M. galloprovincialis* (phylogenetically very closed to *M. edulis* and *M. trossulus*) and *Crassostrea gigas*.

### In vivo cell proliferation

We used the Click-iT EdU Cell Proliferation Kit for Imaging combined with Alexa Fluor 594 dye (invitrogen) to assess the proliferation capacities of MtrBTN2 cells and hemocytes. EdU is incorporated into newly synthesized DNA during cell multiplication. After anesthesia (T_0_), we injected 200 µL of a solution containing 75 µg of EdU and filtered seawater into the posterior adductor muscle of four neoplastic mussels (advanced stage) and four healthy mussels. In addition, one healthy and one cancerous mussel were used as controls and injected with 200 µl of sterile seawater. For the injection, we used a P200 pipette equipped with a 27G needle. EdU injected mussels and controls were then returned to individual tanks with aerated filtered seawater for 9 h. At T_9_, we again anesthetized the EdU-treated and untreated mussels to draw 200 µL of hemolymph from the adductor muscle. The 1 mL syringe was previously filled with 400 µL of formalin to immediately fix the cells and avoid aggregation. After 10 min of incubation, we centrifuged the cellular suspension at 800 × g for 10 min at 4 °C. The pellet was resuspended in 1 mL of PBS + 1% BSA, centrifuged at 1200 × g for 10 min at 4 °C, and washed a second time. We then suspended the pellet in 1 mL of PBS + 0.1% Triton and incubated for 15 min. After incubation, cells were washed two times in PBS + 1% BSA and were ready for EdU detection according to the manufacturer’s recommendations. Nuclei were counterstained in 1 mL of a Hoechst 33,342 (invitrogen) solution 1:2000 in PBS (final concentration 5 µg/mL), washed twice in PBS, and resuspended in 300 µL of PBS. We evaluated cell concentration on a Kova slide and diluted the suspension to a concentration of around 2 × 10^4^ cell/mL to allow counting under an epifluorescence microscope (Axio Imager, ZEISS, 400X magnification) in the successive step. Next, 200 µL of this cell suspension were “cytospun” (Shandon Cytospin 4, Thermo SCIENTIFIC; 10 min, 800 rpm) on SuperFrost slides (Thermo SCIENTIFIC). The multiplication rate in 9 h (expressed as % of circulating cells having incorporated EdU in vivo 9 h after EDU injection) was determined for each individual, observing 10 fields, as the number of Alexa 594-labeled cells (cells that incorporated EdU in the last 9 h)/total number of cells. In addition to allow a count of total nuclei, Hoechst labeling permitted us to discriminate between neoplastic and normal cells according to nucleus size (the DNA content of cancerous cells is at least four times the normal diploid DNA content). We estimated the doubling time (dt) of MtrBTN2 cells. First, we calculated the rate of proliferation (k) according to the exponential growth formula: k = ln(y(t)) / t, where t is he experimental time (9 h) and y is 1 + ((mean % of newly born MtrBTN2 cells) / 100). The % of newly born cells is the number of cells having incorporated EdU in vivo 9 h/2. Then we calculated dt as dt = ln(2)/k.

### Reproductive condition

To assess the reproductive condition of individual mussels, gonadal follicle development and adipogranular (ADG) cell abundance were measured. We examined 16 neoplastic and 18 healthy mussels in various seasons: winter (late January, N = 8), summer (mid-July, N = 13), and late autumn (early December, N = 13). All winter and autumn samples were at an advanced stage of the disease. Five summer samples were at an advanced stage while one was at an early stage and one at a medium stage. We fixed a transversal section of each individual in Davidson's solution for 48 h. These sections were then embedded in paraffin and 3 µM sections were made. Finally, we processed the sections with hematoxylin and eosin staining. We examined tissue sections at X100 and X200 and defined gonadal developmental stages according to Lubet^56^ but modified as follows: stage 0: resting, reconstitution of the energy reserves in connective tissue ADG cells; stage 1, gonia proliferation; stage 2: all developmental stages present with only a few mature gametes; stage 3: for simplification, we grouped Lubet's stages 3A, 3B, and 3C together, having a predominance of mature gametes, low energy reserves, and no or few ADG; stage 4: resorption stage corresponding to Lubet's stage 3D. This classification allowed us to compare the seasonality we observed with the seasonality described in the study of Randriananja^30^ conducted in Pertuis-Breton (Pays de la Loire, Atlantic coast of France). To our knowledge, no data are available for our study area. The sampling sites of both studies are shown in Supplementary Fig. S3. Note that the mussels grown in Agon-Coutainville are initially collected in the Atlantic Coast and are thus descendants of mussels genetically similar to mussels of Pertuis-Breton^57^.

### Statistical analysis

Differences between cancerous and normal cells in terms of survival rate were tested with a Mann–Whitney test performed on the individual daily results of the three wells of the three independent experiments. A Mann–Whitney test was also carried out on proliferation results comparing cancerous cells and hemocytes. For these analyses, the threshold significance level was set at 0.05.

## Supplementary Information

Supplementary Information 1.

Supplementary Information 2.

Supplementary Information 3.

Supplementary Information 4.

Supplementary Information 5.

## Data Availability

All data generated or analysed during this study are included in this published article and its Supplementary Information files.
