# Supplementary figures and images for: Traits of a mussel transmissible cancer are reminiscent of a parasitic life style
Source: Sci Rep. 2021 Dec 16;11:24110. doi: 10.1038/s41598-021-03598-w (PMC8677744; doi:10.1038/s41598-021-03598-w)

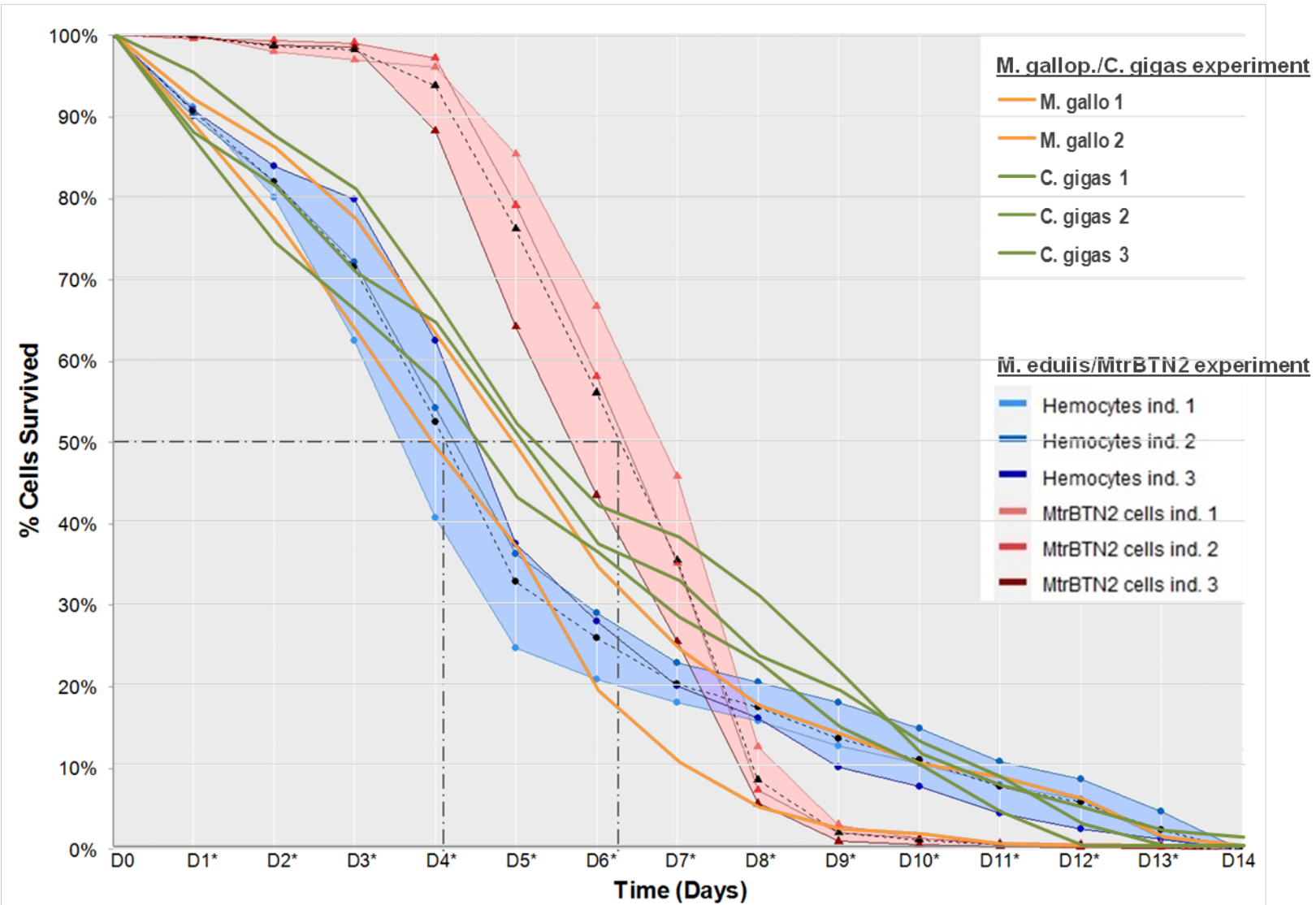

Supplement: Supplementary file 1 — Supplementary Information 1. [file 41598_2021_3598_MOESM1_ESM.pdf]

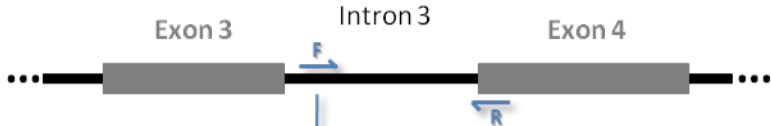

Indel in forward primer

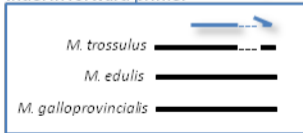

Supplement: Supplementary file 2 — Supplementary Information 2. [file 41598_2021_3598_MOESM2_ESM.pdf]

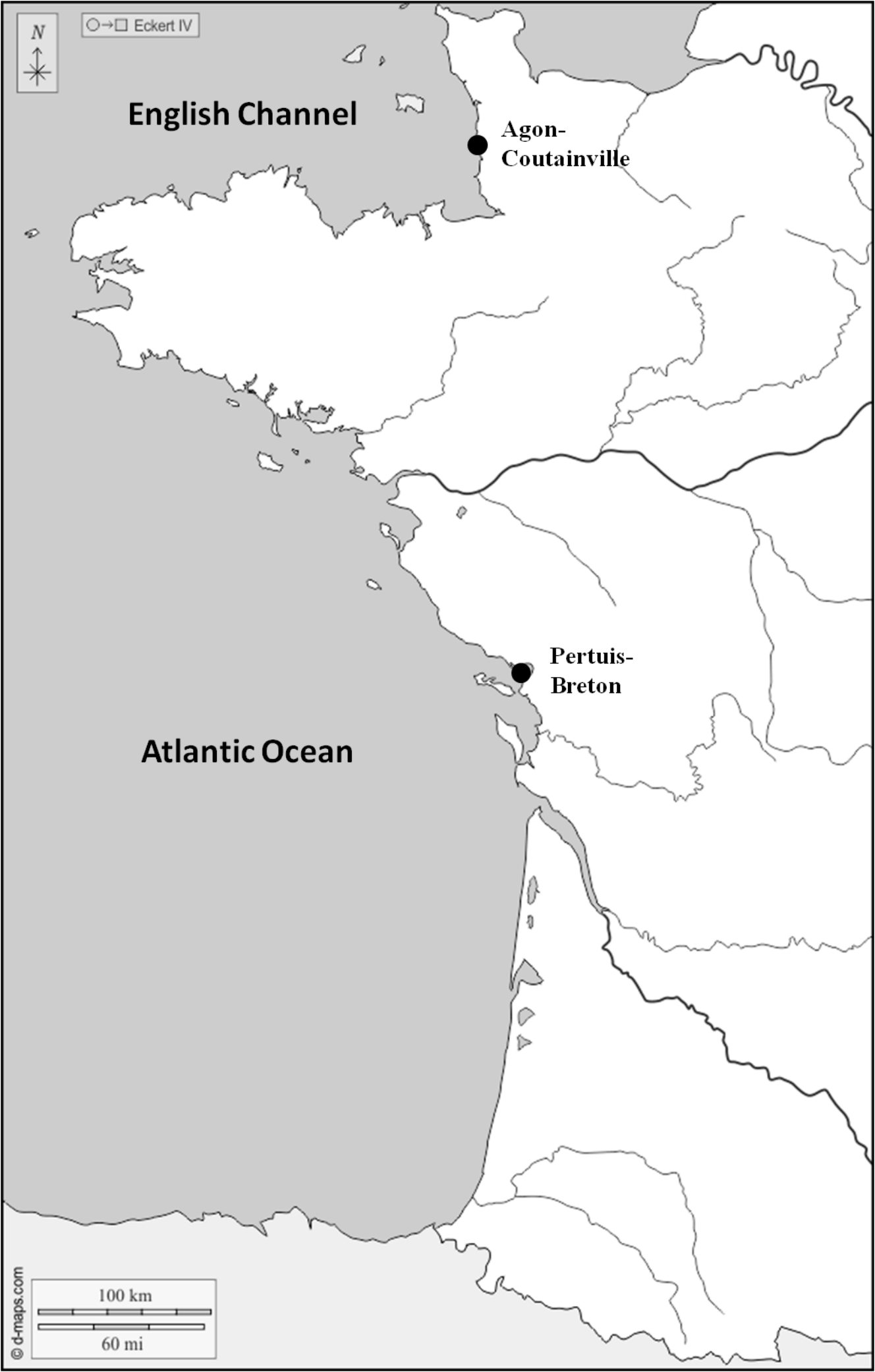

Supplement: Supplementary file 3 — Supplementary Information 3. [file 41598_2021_3598_MOESM3_ESM.pdf]
