# Supplementary material for: Traits of a mussel transmissible cancer are reminiscent of a parasitic life style
Source: Sci Rep. 2021 Dec 16;11:24110. doi: 10.1038/s41598-021-03598-w (PMC8677744; doi:10.1038/s41598-021-03598-w)
Supplement: Supplementary file 4 — Supplementary Information 4. [file 41598_2021_3598_MOESM4_ESM.docx]

Supplementary table S1: results of the MtrBTN2 diagnostic methods (cytology, histology, qPCRs).

Supplementary figure S1: results of the survival tests carried out with *Mytilus galloprovincialis* and *Crassostrea gigas* hemocytes.

Supplementary figure S2: Schematic representation of the Elongation Factor locus targeted by the EF1α-i3 primer pair (F: forward, R: reverse).

Supplementary figure S3: Sampling sites of the mussels used for the gametogenesis studies (original map from <https://d-map.com/>).
